# A complex intervention to support the use of sedative drugs in specialist palliative care: results from the iSedPall pilot study

**DOI:** 10.1186/s12904-026-02112-1

**Published:** 2026-05-05

**Authors:** Christoph Ostgathe, Claudia Bausewein, Eva Schildmann, Maria Heckel, Saskia Kauzner, Carsten Klein, Stefanie Kolmhuber-Seibold, Sabine H. Krauss, Alexander Kremling, Beatrice Odierna, Constanze Rémi, Manuela Schneider, Andreas Seifert, Kerstin Ziegler, Christian Jäger, Jan Schildmann

**Affiliations:** 1 Department of Palliative Medicine, CCC Erlangen – EMN, University Hospital Erlangen, Friedrich-Alexander-Universität Erlangen-Nürnberg (FAU), Erlangen, Germany; 2 Department of Palliative Medicine, LMU University Hospital, LMU Munich, Munich, Germany; 3 Institute for History and Ethics of Medicine, Interdisciplinary Centre for Health Sciences, Martin Luther University Halle-Wittenberg, Halle (Saale), Germany; 4 Department of Criminal Law, Criminal Procedural Law, Commercial Criminal Law and Medical Criminal Law, Friedrich-Alexander-Universität Erlangen-Nürnberg (FAU), Erlangen, Germany; 5 Palliative Medicine , Faculty of Medicine, University of Augsburg, Augsburg, Germany; 6 Paderborn Centre for Educational Research and Teacher Education – PLAZ Professional School, Paderborn University, Paderborn, Germany

**Keywords:** Sedative drugs, Complex intervention, Palliative care, Feasibility, Mixed-methods

## Abstract

**Background:**

International studies show intentional sedation to relieve suffering to be a common and relevant treatment in specialist palliative care. The EAPC framework for using sedative drugs has currently been updated and national recommendations have been disseminated. However, there was a lack of hands-on materials targeted for healthcare professionals and of information materials for patients and informal caregivers to ensure patient-centred care. The iSedPall study group developed a complex intervention to further support the use of sedative drugs in specialist palliative care. The following pilot study aimed at examining the feasibility of the intervention being applied in different settings and by different professions.

**Methods:**

A sequential explanatory mixed-methods design was applied between 02/23 and 01/24. An online survey (pre-post-test) assessed quantitative data on the feasibility of the outcome indicator `confidence in professional skills`, primary feasibility outcomes, and the implementation process. Focus groups added to the quantitative results. Four specialist palliative care services (inpatient and home care) piloted the intervention for nine months.

**Results:**

Global mean scores of primary feasibility outcomes proved the intervention as acceptable, appropriate, and feasible for inpatient and home care settings. The outcome indicator seems to be adequate for measuring changes in healthcare professionals` confidence, especially for physicians. The relevance of the intervention, its impact on practice, and the implementation process have been judged heterogeneously. Promoting (e.g., personal exchange, educational materials) and inhibiting factors (e.g., lack of time and technical resources) for implementation have been stated.

**Conclusions:**

An implementation study would benefit from adaptations regarding the intervention, study design, and implementation strategy. Especially the nursing perspective has to be considered to a greater extent for strengthening the palliative care approach. Context-specific factors seem to play a key role in implementation. Therefore, training of in-group champions, considering local technical and personal resources, and actively engaging the team could mitigate potential barriers and foster the success. Our findings will inform a full-scale implementation study to further explore the use of the intervention by healthcare professionals in clinical practice.

**Trial registration:**

The study was registered in the German Clinical Trials Register (DRKS-ID: DRKS00027241; Date of registration: 10/12/2021; https://www.drks.de/drks_web/setLocale_EN.do).

**Supplementary Information:**

The online version contains supplementary material available at 10.1186/s12904-026-02112-1.

## Background

Over the past decade, a number of international research teams have investigated sedation practice in specialist palliative care (SPC). The Palliative Sedation EU Horizon2020 project for instance has recently examined sedation practice comparing eight European countries with regard to guidelines, medication, and regulations [1]. Based on the results, the EAPC Framework for Palliative Sedation has been revised [2]. The SedPall project explored practice and views of healthcare professionals in Germany [3, 4] and developed based on this expert-approved best practice recommendations for sedative drug use [5]. However, evidence shows continuing heterogeneity in sedation practice related to varying guidelines and terminology [6–8]. The SedPall study group proposed the term `intentional sedation to relieve suffering` to describe the deliberate use of sedative drugs for reducing the consciousness of a patient with refractory symptoms in palliative care [9]. The introduction of this new terminology was a first step in highlighting the full spectrum of using potentially sedative drugs (light to deep, intermittent to until death) and thereby raising more awareness for unintended sedative drug effects.

Given the perceived need to further support best practice of sedative drug use, we set up the project iSedPall (funded by the BMBF: 01GY2020A-C). This project involved the development of a complex intervention to be applied by multidisciplinary healthcare teams in specialist palliative inpatient and home care settings. Hands-on supporting materials, so called `tools`, are provided for medication-related decision-making, information and consent, documentation, and moral challenge analysis, with additional information materials for patients and informal caregivers [10].

According to the German best practice recommendations [5], it was a great concern to address all professions involved in the care of patients for whom the use of sedative drugs is considered or who are currently receiving them, i.e. physicians, nurses, and therapists. Authorized groups (prescribers), in Germany physicians, are responsible for the prescribing of a medication. However, according to literature, the specific role of nurses regarding decisions that do not relate to prescribing medication often remains undefined [11] and a recent scoping review revealed different roles and responsibilities of nurses in intentional sedation depending on the country [12]. All European guidelines analysed in Surges et al. [8] emphasized that „the medical rationale for sedation and the decision-making process should be based on input from a multidisciplinary team, the patient, and family members, rather than by the attending physician alone “ [8]. So, our intervention developed for the use of sedative drugs aimed to take different professional perspectives equally into account [13].

### Aim

The main aim of this pilot study was to examine the feasibility of a complex intervention, its appropriateness, and acceptability as primary feasibility outcomes for different settings in SPC. As a secondary aim, we tested the feasibility of measuring `healthcare professionals` confidence in using sedative drugs` as an outcome indicator for a following implementation study. For exploratory purposes, we collected data regarding the implementation process and potential changes in sedation practice. These initial data will inform the decision on proceeding with a large-scale implementation study.

## Methods

### Study design

The complex intervention was piloted in clinical practice by four pilot centres in inpatient and homecare setting of SPC. During the pilot study taking place between 02/23 and 01/24, a sequential explanatory mixed-methods design was applied [10]. As a first step, an online survey with pre-post-test design assessed quantitative data on the potential outcome indicator, primary feasibility outcomes, and the implementation process to uncover contextual influences and barriers [14]. Afterwards, focus groups were conducted to further explain and add to the quantitative results with qualitative data. Based on the mixed-methods results regarding feasibility of the intervention materials, study design, and implementation strategy, we drew conclusions about the overall feasibility of the intervention to inform the decision on proceeding to a full-scale nationwide implementation study. For reporting of our results, we adhere to the `Consolidated Standards of Reporting Trials (CONSORT) extension for pilot and feasibility trials` [15].

### Intervention

**Fig. 1:**
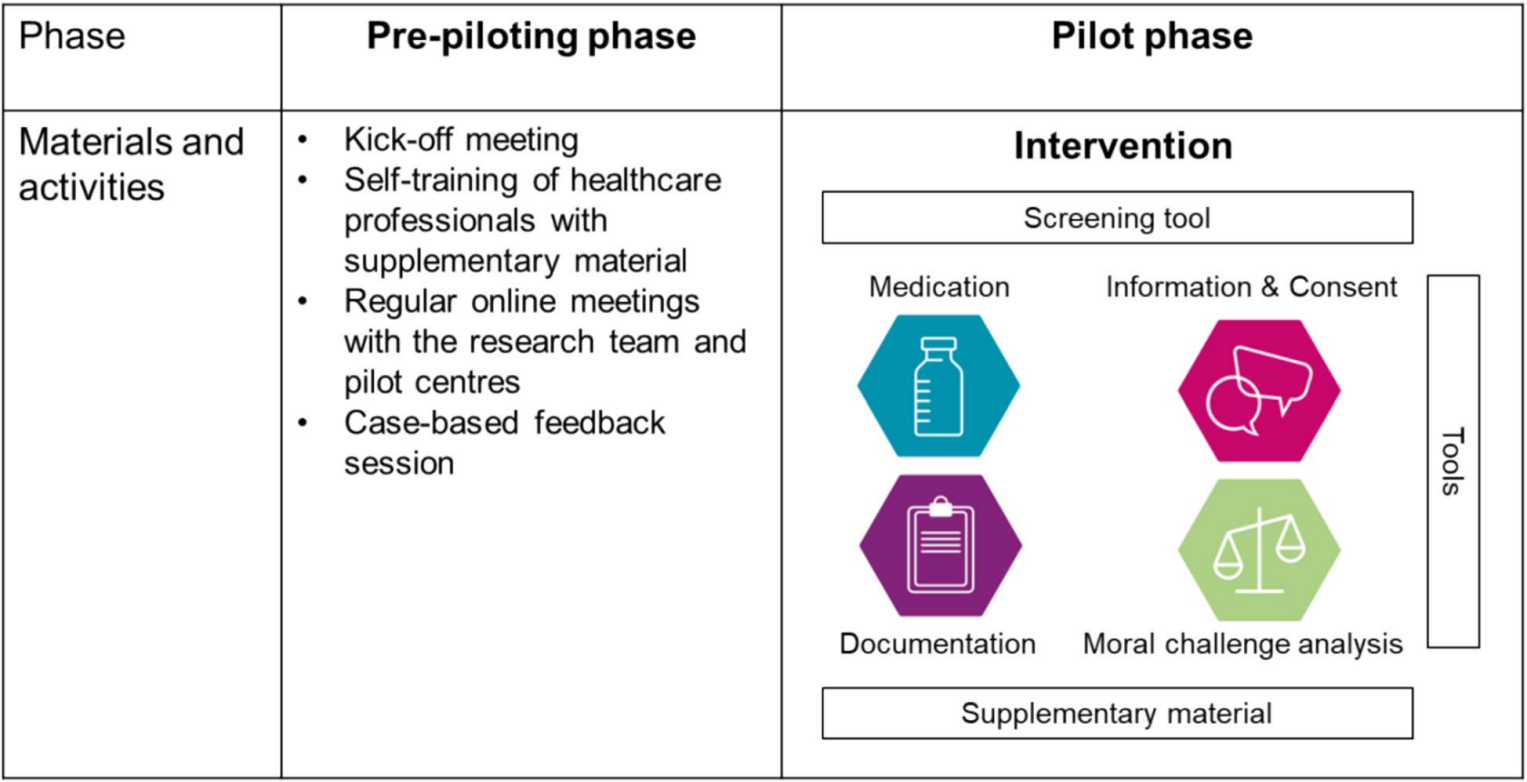
Intervention overview; for more details regarding the tools see https://www.dgpalliativmedizin.de/isedpall

The complex intervention was jointly developed by an interdisciplinary research consortium with active involvement of clinical stakeholders, Patient and Public Involvement (PPI) groups, and (inter-)national advisory board members following the principles of the updated Medical Research Council `Framework for developing and evaluating complex interventions` [14]. The intervention entails I) a screening tool to guide the application of the elements of the intervention in different situations, II) material for medication-related decision-making (e.g., warning list for unintended sedative drug effects), information and consent (e.g., checklist on information provision), documentation (documentation template), and moral challenge analysis (e.g., analyses of ethically challenging situations) – the so called `tools`, III) and supplementary material in form of educational video clips for the healthcare professionals. The intervention also provides additional information material for patients and informal caregivers. Figure 1 shows an intervention overview with the elements of the intervention and pre-piloting activities. More details on the intervention itself according to the `Template for Intervention Description and Replication (TiDieR)` [10] and the development process [13] are reported elsewhere.

### Setting, participants, and implementation

Two German palliative care units and two home care teams, working in rural and urban areas, were recruited by convenience sampling to pilot the intervention and provide feedback over the course of the study by means of regular meetings, workshops, focus groups, questionnaires, and on-site visits. Participants were physicians, nurses, and other professions from the pilot centres. Experience in caring for intentionally sedated patients and sufficient knowledge of German language qualified for study participation. Participants provided written informed consent prior to the study or consented at latest prior to answering the online survey, about which they had been informed before they started the questionnaire. A pre-pilot phase for pre-testing the instruments for data collection and for potential site-specific adaptations of the intervention started in 02/23. The implementation of the intervention in the pre-pilot phase was supported by a kick-off meeting at each site, self-training with videotaped educational materials, and case-based feedback sessions after several weeks of using the intervention for discussing potential barriers of use so far. More details on the implementation during the pre-pilot phase are described elsewhere [13]. The actual nine-month pilot phase took place from 05/23 until 01/24.

### Data collection

#### Quantitative data

A pseudonymised online questionnaire was set up via Unipark and pilot-tested with internal clinical and non-clinical researchers and members of the PPI groups. The revised questionnaire was, together with the supplementary material, disseminated by the pilot centres themselves. A pre-post-test design was applied with measurements before the start of the pre-pilot phase (02/2023 until 03/2023) and after several months of using the intervention (9/2023 until 10/2023). We did not conduct a formal statistical power calculation as it is not recommended for pilot studies [16] but we estimated a 50% response rate for the survey with 95% confidence [10].

The questionnaire consisted of four sections (see below) and concluded with an open-ended question for additional feedback. As control question, we asked if and which tool(s) have been used. See supplementary 1 for the detailed questionnaire.

##### Participants` characteristics

Sociodemographic data (profession, setting, years of professional activity in SPC, age), experience with intentional sedation (notably regarding indication, conduction or monitoring), and the acquaintance of the national best practice recommendations were assessed.

#### Outcome indicator

The feasibility of the `healthcare professionals` confidence in the use of sedative drugs` as potential outcome indicator to assess changes over the course was tested with a pre-post-test design whereby initial data for a future sample size calculation were collected [10]. Based on the Health Professionals Competence Scales (HePCos) [17], seven sedation-specific situations in SPC dealing with medication-related decisions, information and consent, documentation, and ethically challenging situations were self-developed and slightly adapted for physicians as well as for nurses and other professions. The level of confidence was examined on a 4-point Likert scale (0 = `not confident at all`; 3 = `very confident`).

The following variables were added only for the post-test survey. The items assessing participants` characteristics and the outcome indicator remained the same for both measurements.

#### Primary feasibility outcomes

We drew on the implementation outcome measures `acceptability, appropriateness, and feasibility` as defined by Proctor et al. [18]. Therefore, we applied the German version of the Acceptability of Intervention Measure (AIM), Intervention Appropriateness Measure (IAM), and Feasibility of Intervention Measure (FIM) [19], validated by Kien et al. [19, 20]. Each scale consists of four items rated on a 5-point Likert scale (1 = `completely disagree`; 5 = `completely agree`).

#### Process evaluation

16 Likert scale statements (1 = `completely disagree`; 5 = `completely agree`, with `no opinion` option) were formulated based on the Consolidated Framework for Implementation Research (CFIR) covering the domains `culture`, `implementation climate`, and `readiness for implementation` [21]. These factors are expected to influence the implementation of research results. The items were primarily examined to uncover insights regarding the implementation of the intervention into existing systems, the potential impact on practice, and the perceived need for the intervention. One open-ended question regarding potential facilitating factors for usage has been added at the end.

#### Qualitative data

We conducted centre and setting specific focus group discussions with healthcare professionals (physicians, nurses, other professions) from all pilot centres. We invited the leading physicians and nurses form the pilot centres to participate, and asked them to recruit further staff members from their teams who had used the intervention to take part in the discussions. Some participants took part in both centre and setting specific focus groups.

As the focus group discussions took place after the quantitative data collection phase was completed (10/23 until 01/24), we were able to focus on topics that were either not part of the online survey or appeared as striking with regard to participants’ answers, e.g., differences between professions. The discussion guide questions were informed by the CFIR [21] and focused on different dimensions of feasibility, e.g., acceptability, user-friendliness and relevance of the intervention, general user experience and need for adaptation, facilitators and barriers to use, and potential unintended consequences. See supplementary 2 for the detailed discussion guide.

### Data analysis

#### Quantitative analysis

We used IBM SPSS Statistics 28.0. for statistical analyses. An interim analysis was run with initial data after the pre-test measurement (during pre-pilot phase). Descriptive statistics (e.g., means, medians, standard deviations, frequencies and percentages) were calculated for participants` characteristics, process evaluation items, and frequency of the use of the tools. For analysing the healthcare professionals` confidence and primary feasibility outcomes (AIM, IAM, FIM), global scale scores were calculated by averaging the items for each scale. Reliability scores (Cronbach`s alpha) were provided for each scale. Potential changes in the healthcare professionals` confidence in using sedative drugs were assessed by comparing data between professions for both measurements. Due to the relatively small sample size and the non-normal distribution for the confidence scores, we decided on calculating the medians [22]. As the assumption for normal distribution and variance homogeneity was confirmed, independent samples *t*-tests were conducted to compare settings and professions in terms of feasibility outcomes. A significance level of *p* < 0.05 was determined. The answers regarding the open-ended questions were thematically structured as part of process evaluation.

#### Qualitative analysis

The focus group interviews were transcribed according to Dresing & Pehl [23] and analysed in accordance to qualitative content analysis as described by Schreier [24] using MAXQDA version 2022 [25]. The analysis was divided into two phases: 1) designing and finalizing the code system, initially based on the discussion guide and inductively adjusted, in combination with coding by two scientific employees to ensure completeness of coding and inter-coder-reliability; 2) tool-specific analysis in terms of required adjustments and topic-related analysis in terms of acceptability, relevance, and user-friendliness as dimensions of feasibility; as well as exploring the implementation process with regard to promoting and inhibiting factors, unintended consequences, experienced impact of the educational materials, and changes and persistence of practice since using the intervention.

#### Overall feasibility

Quantitative and qualitative data were separately analysed and merged afterwards as presented in the results section. Quantitative data of the AIM, IAM, and FIM [20] served as main criteria as predefined in our Study Protocol [10]. Mean measure scores ≥ 3 were taken to indicate a favourable outcome in terms of feasibility, acceptability, and appropriateness according to common evaluations of newly developed interventions [26, 27]. Further feedback regarding the intervention and the implementation process was provided by the pilot centres over the course of the study, which helped to understand the field, and informed the evaluation of overall feasibility. The mixed-methods results were discussed within the research consortium to draw conclusions on overall feasibility with implications for proceeding to an implementation study.

## Results

### Participants

In total, *n* = 100 professionals from the four pilot centres formed the potential survey cohort by receiving the weblink for the online survey. We had a response rate of 49% for the pre-test measurement and of 33% for the post-test measurement, whereby the former is in line with our previously estimated response rate [10]. Regarding the pre-test, *n* = 13 physicians, *n* = 32 nurses and four persons from other professions participated. In the post-test, especially the response rate for the nursing (*n* = 17) and homecare (*n* = 12) staff decreased, while for physicians it remained the same (*n* = 9). Seven professionals participated in both surveys.

We conducted six focus group discussions (one per pilot centre, two per setting), with 3 to 8 participants each (physicians, nurses, and other professions) (see Table 1). Data saturation was reached.

**Table 1:** Participants` characteristics

Survey cohort				
	**pre-test** respondents *n* = 49		**post-test** respondents *n* = 33	
profession				
physicians	13	(26.5%)	9	(27.3%)
nurses	32	(65.3%)	17	(51.5%)
other professions	4	(8.2%)	7	(21.2%)
setting				
inpatient	25	(51.0%)	21	(63.6%)
homecare	24	(49.0%)	12	(36.4%)
professional activity in specialist palliative care				
< 1 year	9	(18.4%)	4	(12.1%)
1–4 years	9	(18.4%)	4	(12.1%)
≥ 5 years	31	(63.3%)	25	(75.8%)
age groups				
16–25 years	4	(8.2%)	1	(3.0%)
26–35 years	3	(6.1%)	5	(15.2%)
36–45 years	11	(22.4%)	6	(18.2%)
46–55 years	14	(28.6%)	8	(24.2%)
≥ 56 years	17	(34.7%)	13	(39.4%)
popularity of best practice recommendations *did you read the national recommendations*?				
physicians (*n* = 13)				
yes	11	(84.6%*)	9	(100%*)
no	2	(15.4%*)	0	(0%*)
nurses & other professions (n = 36)				
yes	17	(47.2%**)	17	(70.8%**)
no	19	(59.4%**)	5	(20.8%**)
missing	0	(0%*)	2	(8.3%**)
experience with intentional sedation				
* I am aware of when an intentional sedation is indicated*				
physicians				
does not apply & rather does not apply	0	(0%*)	0	(0%*)
indifferent	3	(23.1%*)	0	(0%*)
rather applies & fully applies	10	(76.9%*)	9	(100%*)
nurses & other professions				
does not apply & rather does not apply	4	(11.1%**)	1	(4.2%**)
indifferent	13	(36.1%**)	9	(37.5%**)
rather applies & fully applies	19	(52.8%**)	12	(50.0%**)
missing	0	(0%*)	2	(8.2%**)
* I have enough experience to conduct (physicians)/monitor (nurses) an intentional sedation*				
physicians				
does not apply & rather does not apply	1	(7.7%*)	0	(0%*)
indifferent	2	(15.4%*)	0	(0%*)
rather applies & fully applies	10	(76.9%*)	9	(100%*)
nurses & other professions				
does not apply & rather does not apply	6	(16.7%**)	4	(16.7%**)
indifferent	11	(30.6%**)	3	(12.5%**)
rather applies & fully applies	19	(52.8%**)	15	(62.5%**)
missing	0	(0%**)	2	(8.2%**)

**Focus group interviews cohort**				
	**Centre specific** (*n* = 4)		**Setting specific** (*n* = 2)	
profession				
physicians	9		6	
nurses	7		4	
other professions	2		2	
professional activity in specialist palliative care, grouped in years				
< 1 year	1		2	
1–4 years	4		2	
≥ 5 years	13		8	
age groups in years				
16–25 years	0		1	
26–35 years	3		2	
36–45 years	6		2	
46–55 years	4		4	
≥ 56 years	5		3	

* = of physicians; ** = of nurses and other professions

### Scope of the tools` usage

Within all valid responses (n = 28), 22 staff members (78.6%) did use at least one tool of the intervention during the study period. Comparing the settings, 12 participants (70.6%) of the inpatient setting and 10 participants (90.9%) of the home care setting did use at least one tool. Regarding professions, nine physicians (100%) and 10 nurses (71.4%) used at least one tool.

### Outcome indicator (Healthcare professionals` confidence)

The reliability analysis proved the internal consistency of the scales for physicians and nurses and other professions (*n* = 7 items each), with Cronbach`s alpha ranging from 0.86 to 0.92. For physicians, descriptive data showed a slight tendency for increased confidence scores from pre-test (*Md*_1_ = 1.83 (95%-CI: [1.50; 1.83]), *SD* = 0.47, *n* = 13) to post-test measurement (*Md*_2_ = 2.33 (95%-CI: [2.14; 3.00]), *SD* = 0.37, *n* = 9). The reported change only reflects group-level differences between samples rather than within-person change. For nurses and other professions, there were no apparent changes from pre-test (*Md*_1_ = 2.00 (95%-CI: [1.57; 2.14]), *SD* = 0.65, *n* = 33, missing: *n* = 3) to post-test measurement (*Md*_*2*_ = 2.00 (95%-CI: [1.86; 2.52]), *SD* = 0.51, *n* = 22, missing: *n* = 2).

These quantitative results are related to different perspectives of physicians and nurses on the intervention and their responsibilities in sedation practice which have been revealed through the focus group interviews. Exemplary of the attitudes of several nurses interviewed, it has been expressed that the intervention is aimed at physicians as they are responsible for all medical aspects:

*“So, for us as nurses, we don't need a tool anyway. [...] Because this medical responsibility and the medication is your responsibility.” (4/370–372)*

### Primary feasibility outcomes

#### Acceptability, appropriateness and feasibility

In the survey, 27 participants (81.8%) answered all questions (missing values: *n* = 6; 18.2%) related to feasibility outcomes. The reliability analysis of the scales (*n* = 12 items) yielded Cronbach`s alpha ranging from 0.89 to 0.92 which are in line with those of the validity study [19]. The quantitative data revealed satisfying global scores with *M* = 3.42 (*SD* = 0.94) for acceptability, *M* = 3.50 (*SD* = 0.17) for appropriateness, and *M* = 3.36 (*SD* = 0.17) for feasibility in line with our predefined criteria [10]. An independent samples *t*-Test revealed significant differences in terms of acceptability (*t*(20) = 2.37, *p* = 0.028) and appropriateness (*t*(20) = 2.16, *p* = 0.043) between physicians and nurses meaning that physicians scored significantly higher for acceptability and appropriateness. Other professions were not part of this comparison since they had too many missing values. No significant differences were found between settings. Figure 1 presents a comparison between global mean scores for physicians and nurses.

**Fig. 2:**
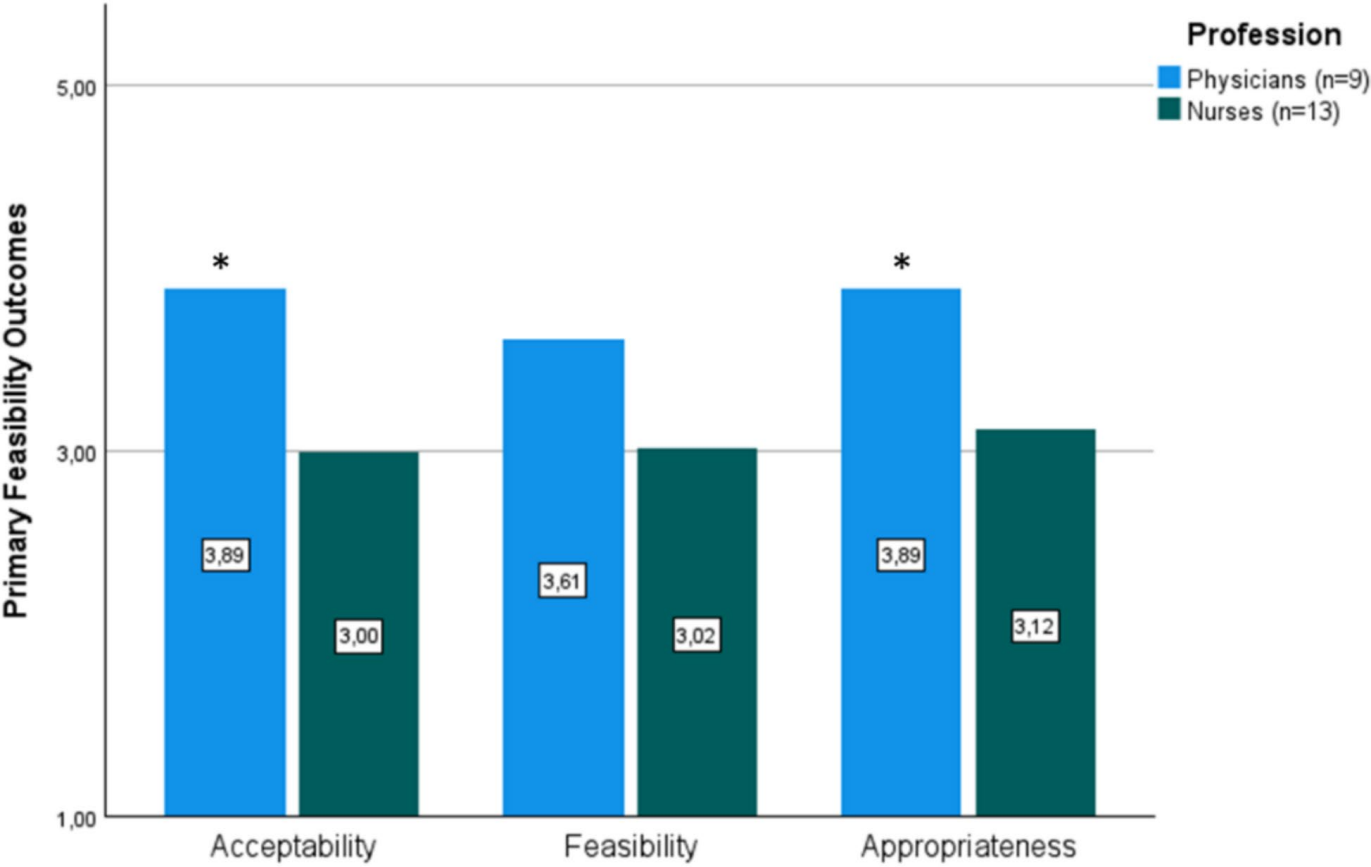
Mean scores of primary feasibility outcomes compared for physicians and nurses

In the qualitative data, feasibility was reflected within the codes `acceptability`, `user-friendliness`, and `relevance`. Both promoting and inhibiting factors were identified (Fig. 2).

#### Acceptability

Participants seemed to accept the intervention, expressing that it could support their daily work, be helpful for patients and for a legal safeguarding. A physician in a leading position said:

*"Because I am always worried that we use medication WELL-meaning for symptom control but achieve an effect which is then called sedation and which possibly does not comply with the will of the patient or the general agreement – and thus contradicts it. That's why I think the study is good so far. " (1/37)*

As inhibiting factors, the lack of time in daily practice and the amount of effort needed to get familiar with the materials were mentioned.

#### User-friendliness

Participants emphasized the comprehensibility of the materials, its supportive character, and the usefulness of the materials for training of new employees. A physician in a leading position stated:

*"At least, we can use it for training when we are getting a new ward physician again, until it becomes routine." (5/113)*

As inhibiting factors, the high expenditure of time for introduction, the quantity and complexity of the material, the additional workload, and difficulties regarding the integration into their work processes were mentioned by participants.

A physician in a leading position argued in regards to the complexity of the material:

*“But it’s confusing. And it’s also unclear, when what. And it’s also unclear, what is necessary, what is optional.” (2/229)*

#### Relevance

There is seen a need for the intervention, even though it has been judged heterogeneously. According to quantitative data, 15 participants (62.5%; n = 24) would like to use the intervention after the study ends. These participants were 7 physicians (inpatient: n = 3), 6 nurses (inpatient: n = 3) and 2 participants of other professions (inpatient: n = 1) of both settings with all levels of professional experience, whereas most of them (n = 10) had at least 5 years of professional experience.

In the focus groups, a physician in a leading position stated that there was a general need for such an intervention:

*"We have done within the study what I thought we would need in the palliative care unit anyway.” (2/256)*

Furthermore, the intervention was seen as helpful to avoid wrong medical treatment, as containing relevant professional knowledge, and as guiding work in general. A nurse in a leading position said:

*"When we prepare a […] planned intended sedation practically, […] WHAT can be packed in there or what HAS to be in there? And this guide or toolbox naturally provided us with support in this regard." (6/36)*

As negative comments regarding the relevance of the intervention, professionals expressed that they themselves have sufficient professional experience already, that the material is not suitable and there is no need for such an intervention. Some participants complained that using the material does not always lead to an improvement for patients and their relatives. Therefore, the additional effort could be seen as a burden instead of a support for the daily work. For instance, a physician stated:

*"And when an intervention such as this one, in most cases, does NOT REALLY lead to a consequence of action and does not do the patient some good, then I develop the stress to say: Now I am also endangering other patients. Because I am talking about a situation where I ALWAYS have to prioritize." (1/184)*

### Process evaluation

#### Implementation process

In the survey, 30 participants (90.9%) answered the statements (missing values: *n* = 3; 9.1%). Participants with `no opinion` answer were deleted in the respective analysis. For 10 respondents (41.7%; *n* = 24), the implementation of the intervention into existing routines and practices was successful whereas seven respondents (29.2%) did not agree on that. According to 16 respondents (69.6%; *n* = 23), the intervention is used by default when it comes to using potential sedative drugs. The supplementary material helped 19 participants (70.4%; *n* = 27) to understand the aim and content of the intervention. See supplementary file 3 for the detailed frequency distribution of responses.

#### Promoting and inhibiting factors during implementation

In line with quantitative data, the focus group interviews revealed that the educational material and personal exchange with the study team was supportive. Participants also expressed that an integration into the team meetings might be helpful. For instance, a physician stated:

*“I think that for all planned situations where we act calmly, interdisciplinary, in the context of meetings, this is all very useful and a great help.” (1/61)*

With regard to inhibiting factors, participants pointed to their lack of time for a reasonable implementation and criticized the lack of a clear explanation of the aims of the study and practical examples as well as a profession specific orientation. This applies to the educational material as well, especially the duration of the videos and the poor quality of the video design have been criticized.

A physician in a leading position made the following suggestions for the educational materials:

*“Example cases might be better. So that you have one case where it's only about symptom control [...] One with intensive, intentional sedation [...] These are examples. And where you can then see what can I possibly use and when is it not at all expected that I use something?” (2/33)*

Healthcare professionals also referred to difficult conditions within their institution (e.g., technical problems, staff shortage and change of personnel) and argued that the accompanying research tasks were even more stressful.

Lack of staff, time, and technical equipment as well as IT constraints led to a delayed intervention start. These conditions particularly affected self-training via video clips, which was sometimes done after hours. A physician in a leading position described:

*"If we watch these instruction videos and then we sit there after the official team meeting, there were five, six people sitting there in their FREE time." (2/235)*

In conclusion, the educational material and the personal interaction with the study team were identified as the main facilitators during implementation, while staff shortages, time constraints and IT issues were the main barriers. Having technical equipment that works properly is a practical requirement for successful implementation.

#### Changes in practice

According to quantitative data, seven participants (30.4%; *n* = 23) reported perceived changes in sedation practice in their institution after using the intervention. Otherwise, 10 respondents (43.5%; *n* = 23) did see no impact on sedation practice.

Participants of the focus groups stated that they observed positive changes in practice. This included more attention for handling potentially sedative drugs, a better understanding of sedation and therefore an improvement of practice, better exchange within the team as well as better communication with patients and their relatives. With regard to negative or no changes, some argued that they had observed no changes of practice in general, in case of emergency as well as of sedation concept, time expenditure, and workload. As one nurse in a leading position encapsulated it:

I think it still has to get into the heads.“ (1/260)

### Overall feasibility

#### Intervention

The intervention is feasible, acceptable, and appropriate for using sedative drugs and intentional sedation in inpatient and home care settings, especially from the physicians` perspective. It can be integrated into clinical systems even though the implementation process means much time effort. The elements of the intervention appear to be complex and extensive. It turned out that some elements were continuously used while others were more useful for educational purposes. Nurses reported no relevant changes in confidence level after using the intervention. The educational videos have been criticized for their duration, their poor design, and lack of case specificity.

### Study design

The selected outcome indicator is feasible and appears to be reliable and valid, especially for physicians, as most of the surveyed physicians indicated to encounter the presented situations in their professional activity. However, for nurses and other professions, the selected situations were at least for some of the respondents not part of current professional activity. Given the response rates for the pre-post-test survey, we reached the expected sample size of 50% [10] at least for the first measurement.

## Discussion

### Main findings

We have developed and piloted a first complex intervention to provide support for best practice use of sedative drugs in SPC. After piloting, we draw the following conclusions for the design of a future implementation study including necessary adaptations regarding the intervention, improvements of implementation strategy, and study design. The elements of the intervention need to be adapted to be more user-centred and reduced in quantity. Additionally, a recommendation for the intended use of the elements would be helpful. The implementation in healthcare practice must be appropriately planned and supported afterwards. Therefore, local facilitators with sufficient knowledge of team structures and technical infrastructure should support implementation on-site. The educational material has to be reworked and set up in a targeted way by involving educational and technical competencies. In general, the local conditions regarding technical infrastructure should be considered in more detail to guarantee smooth access to online-based study materials (e.g., questionnaires). The initial results will provide essential data on variability within the sample and will allow for a power analysis to determine the required sample size and effect size to be expected for a larger study.

The fact that physicians` and nurses` assessment of the intervention`s acceptability, appropriateness, and impact was quite different, can be seen as a key finding of the pilot study and provides valuable information for future adaptations of the intervention. As nurses felt rather less addressed by the intervention materials, the nursing perspective has to be considered to a greater extent within the materials. Perhaps additional tools specifically addressing nursing tasks e.g., supportive conversations with patients and family members during the decision-making process, would be a beneficial supplement to emphasize the role of nurses during intentional sedation. It was striking that the response rate for the nursing staff decreased for the post-test measurement. Perhaps, this is connected to the unchanged confidence level over the course for nurses according to descriptive statistics. However, it might also be the case that nurses could not identify their role in the presented situations of the outcome measurement and therefore, potential changes in confidence may not have been assessed. So, this outcome indicator appears to reflect physicians` roles and responsibilities better than those of nurses and other professions. Nevertheless, results have to be interpreted with caution due to the small sample sizes.

As already mentioned at the beginning, the professional role understanding within the sedation process is not clearly defined. As shown by our data as well, intentional sedation sometimes still seems to be treated as sole responsibility of physicians. In contrast, the key role of nurses in decision-making, implementation, monitoring, information sharing, and care for the patient and the relatives has currently been highlighted [12]. Future work should therefore aim to further strengthening palliative care, including intentional sedation as a multi-professional team approach and should emphasise the role of nurses.

There were no differences between settings in terms of feasibility outcomes showing that the intervention appears to be suitable for both inpatient and homecare settings.

Several participants perceived changes in practice since the intervention has been implemented. Interpreted with caution due to the pilot study design, this allows for an outlook in terms of potential positive effects on clinical practice by using the intervention. On the other hand, almost half of the participants did not see any impact on sedation practice, which may be related to the relatively high baseline level of professional confidence, self-reported experience in conducting or monitoring intentional sedation, and average years of professional experience of the participants. This appears to be also reflected in the perceived relevance and need for the intervention which were ambivalent.

We have taken `culture`, `implementation climate`, and `readiness for implementation` into account within the survey as they all seem to play a significant role in successful change processes [21]. Our results show that especially `readiness for implementation` consisting of the subconstructs `available resources, leadership engagement and access to information and knowledge` was key to successful implementation in a healthcare setting with limited resources and tight schedules. Whereas `culture` and `implementation climate` have the potential to foster the team`s awareness and readiness for change, we experienced those constructs to have minor influence on our implementation process. Many research teams have explored the phenomenon of successful changes with special focus on healthcare settings as they constantly experience changes due to technical and medical progress as well as social and political changes [28]. Therefore, three categories for successful change have been identified, namely the opportunity to influence the change, being prepared for the change, and valuing the change [28]. In combination with our results, these points should be seriously considered before implementing a new initiative to increase the likelihood of success.

### Strengths and limitations

As a strength of our pilot study, we used a multicentre design with a multidisciplinary research consortium in collaboration with a PPI group, the intervention taking both settings and different professions into account, and the use of a mixed-methods approach. Patient and public participation, which was already applied in the development phase, continued to facilitate the development of data collection instruments and the interpretation of results.

There are some limitations regarding the pre-post-test survey. Getting access to the online questionnaire was sometimes difficult for staff members due to IT constraints and lack of technical resources. Therefore, we cannot be sure if we have reached out to everyone we wanted to. The response rate for the post-test measurement decreased, which could affect the accuracy of effect size estimation for a larger study. In addition, only seven participants took part in both measurements. A future study should develop a strategy for ensuring participation in both measurements to assess within-subject effects. The feasibility of the outcome indicator has not been proved by statistical means and we were not able to prove construct validity, as we did not find a comparable instrument for assessing professional confidence in our context. So maybe a validation study for the outcome indicator were to provide the final proof.

Regarding the implementation process, the healthcare professionals of the pilot centres reported, that sometimes it has not been easy to clearly differentiate between the intervention itself and additional tasks for study purposes, which have been perceived quite exhausting. For that reason, the summative evaluation of the intervention could entail a negative bias.

As a deviation from our study protocol, we were not able to conduct interviews with patients and/or informal caregivers to whom the materials were given but we respected the extremely sensitive and existential situation for those families. Instead, we had access to non-involved patients and informal caregivers, with whom we talked about their sedation experiences and explored how our materials could have helped which is not reported here.

### Implications

Our findings will inform the future planning of a full-scale implementation study to further explore possible benefits for healthcare professionals using the intervention in different palliative care settings. The piloted elements of the intervention are now publicly available, also appropriate to be used for palliative care education. Future studies should examine how the intervention should be adjusted according to different languages, cultures and religious beliefs, for instance differences in dealing with death within goals of care discussions [29].

With our study, we promoted the preliminary developed recommendations on best practice use of sedative drugs which apparently increased their acquaintance within the pilot centres. By supporting best practice of sedative drug use, we aim to strengthen the healthcare professionals` confidence regarding their professional skills for delivering patient-centred care.

In recent years, the number of published pilot and feasibility studies has continuously increased. The literature suggests varying approaches for defining and constituting pilot studies, which may affects comparability and quality of research [30, 31]. The same applies to strategies for evaluating feasibility studies [31, 32]. By sharing relevant findings and challenges from our pilot study, we build upon current literature on pilot and feasibility studies and contribute to the development in this area.

## Conclusion

Clinical practice and the terminology for intentional sedation in SPC is quite heterogeneous, despite the existence of numerous guidelines for sedative drug use. Best practice recommendations have been published for Germany, but there was still a lack of hands-on materials for healthcare professionals, patients, and informal caregivers. We filled this gap by developing and piloting an intervention to support best practice use of sedative drugs. The findings of our pilot study proved the intervention to be feasible, acceptable, and appropriate for both inpatient and homecare settings, whereas physicians seemed to feel more addressed by the materials than nurses. Minor adaptations for improvement have been concluded for a future study. Among other things, the perspective of nurses should be engaged more intensively and the implementation of the initiative on-site should be thoughtfully prepared closely supported.

## Supplementary Information

Supplementary Material 1.

Supplementary Material 2.

Supplementary Material 3.

Supplementary Material 4.

## Data Availability

The datasets generated and/or analysed during the current study are not publicly available due to data protection reasons. For more details, please contact christoph.ostgathe@uk-erlangen.de.

## Abbreviations

AIM: Acceptability of Intervention Measure
CFIR: Consolidated Framework for Implementation Research
CONSORT: Consolidated Standards of Reporting Trials (extension for pilot and feasibility trials)
DGP: German Association for Palliative Care
EAPC: European Association for Palliative Care
FIM: Feasibility of Intervention Measure
HePCos: Health Professionals Competence Scales
IAM: Intervention Appropriateness Measure
PPI: Patient and Public Involvement
SPC: Specialist palliative care
TiDieR: Template for Intervention Description and Replication

## References

[1] Payne SA, Hasselaar J. European Palliative Sedation Project. J Palliat Med Februar. 2020;23(2):154–5. 10.1089/jpm.2019.0606 PMC7138599 32023195

[2] Surges SM, Brunsch H, Jaspers B, Apostolidis K, Cardone A, Centeno C, et al. Revised European Association for Palliative Care (EAPC) recommended framework on palliative sedation: An international Delphi study. Palliat Med Februar. 2024;38(2):213–28. 10.1177/02692163231220225 PMC10865771 38297460

[3] Meesters S, Bazata J, Handtke V, Gehrmann J, Kurkowski S, Klein C, Bausewein C, Schildmann E, for the SedPall Study Group. “It’s pretty much flying blind in the home care setting”: A qualitative study on the influence of home care specific circumstances on sedation in specialist palliative home care. Palliat Med. 2023;37(1):140-148. doi: 10.1177/02692163221128938. 10.1177/02692163221128938 PMC9841818 36242514

[4] Bazata J, Meesters S, Bozzaro C, Handtke V, Schildmann J, Heckel M, Ostgathe C, Bausewein C, Schildmann E, SedPall SG. An easier way to die?-A qualitative interview study on specialist palliative care team members’ views on dying under sedation. Palliat Med. 2025.Palliat Med. 2025 ;39(4):517-526. doi: 10.1177/02692163251321320. 10.1177/02692163251321320 PMC11977801 39981842

[5] Ostgathe C, Bausewein C, Schildmann E, Bazata J, Handtke V, Heckel M, et al. Expert-approved best practice recommendations on the use of sedative drugs and intentional sedation in specialist palliative care (SedPall). BMC Palliat Care. 2023;22(1):126. doi: 10.1186/s12904-023-01243-z. 37667303 10.1186/s12904-023-01243-z PMC10476406

[6] Kremling A, Schildmann J. What do you mean by „palliative sedation“? BMC Palliat Care. 2020;19(1). 10.1186/s12904-020-00635-9 PMC7513316 32967659

[7] Klein C, Voss R, Ostgathe C, Schildmann JA, SEDPALL study group. Sedation in palliative care—a clinically oriented overview of guidelines and treatment recommendations. Dtsch Arztebl Int. 2023;120(14):235–42. 36851822 10.3238/arztebl.m2023.0034 PMC10282508

[8] Surges SM, Garralda E, Jaspers B, Brunsch H, Rijpstra M, Hasselaar J, et al. Review of European guidelines on palliative sedation: a foundation for the updating of the European Association for Palliative Care framework. J Palliat Med. 2022;25(11):1721–31. 35849746 10.1089/jpm.2021.0646

[9] Kremling A, Bausewein C, Klein C, Schildmann E, Ostgathe C, Ziegler K, et al. Intentional Sedation as a Means to Ease Suffering: A Systematically Constructed Terminology for Sedation in Palliative Care. J Palliat Med Mai. 2022;25(5):793–6. 10.1089/jpm.2021.0428 PMC9081045 35073180

[10] Ostgathe C, Bausewein C, Schildmann E, Bazata J, Heckel M, Kauzner S, et al. Use of sedative drugs in specialist palliative care (iSedPall): a multi-modal intervention pilot study protocol. Pilot Feasibility Stud. 2025;11(1):45. 40211291 10.1186/s40814-025-01627-3 PMC11984285

[11] Cherny NI, Radbruch L, The Board of the European Association for Palliative Care. European Association for Palliative Care (EAPC) recommended framework for the use of sedation in palliative care. Palliat Med. 2009;23(7):581–93. 19858355 10.1177/0269216309107024

[12] Heino L, Stolt M, Haavisto E. The practices and attitudes of nurses regarding palliative sedation: A scoping review. Int J Nurs Stud Mai. 2021;117:103859. 10.1016/j.ijnurstu.2020.103859 33545642

[13] Kauzner S, Schneider M, Heckel M, Klein C, Bausewein C, Schildmann E, et al. Development of a complex intervention to support the use of sedative drugs in specialist palliative care (iSedPall). Palliat Med Rep. 2024;5(1):527–36. 40012852 10.1089/pmr.2024.0042 PMC11864855

[14] Skivington K, Matthews L, Simpson SA, Craig P, Baird J, Blazeby JM, Boyd KA, Craig N, French DP, McIntosh E, Petticrew M, Rycroft-Malone J, White M, Moore L. A new framework for developing and evaluating complex interventions: update of Medical Research Council guidance. BMJ. 2021 Sep 30;374:n2061. doi: 10.1136/bmj.n2061. 10.1136/bmj.n2061 PMC8482308 34593508

[15] Eldridge SM, Chan CL, Campbell MJ, Bond CM, Hopewell S, Thabane L, Lancaster GA. CONSORT 2010 statement: extension to randomised pilot and feasibility trials. BMJ. 2016;355:i5239. doi: 10.1136/bmj.i5239. 10.1136/bmj.i5239 PMC5076380 27777223

[16] Lancaster GA, Dodd S, Williamson PR. Design and analysis of pilot studies: recommendations for good practice. J Eval Clin Pract Mai. 2004;10(2):307–12. 10.1111/j..2002.384.doc.x 15189396

[17] Grebe, Christian, Schürmann, Mirko, Latteck, Änne-Dörte. Health Professionals Competence Scales (HePCoS) - Startseite. Health Professionals Competence Scales (HePCoS)PCOS.. Verfügbar unter: https://www.hepcos.com/cms/. Zitiert 13. Juli 2023.

[18] Proctor E, Silmere H, Raghavan R, Hovmand P, Aarons G, Bunger A, et al. Outcomes for implementation research: conceptual distinctions, measurement challenges, and research agenda. Adm Policy Ment Health Ment Health Serv Res. 2011;38(2):65–76. 10.1007/s10488-010-0319-7 PMC3068522 20957426

[19] Weiner BJ, Lewis CC, Stanick C, Powell BJ, Dorsey CN, Clary AS, et al. Psychometric assessment of three newly developed implementation outcome measures. Implement Sci. 2017;12(1):108. 28851459 10.1186/s13012-017-0635-3 PMC5576104

[20] Kien C, Griebler U, Schultes MT, Thaler KJ, Stamm T. Psychometric testing of the German versions of three implementation outcome measures. Glob Implement Res Appl. 2021;1(3):183–94.

[21] Damschroder LJ, Aron DC, Keith RE, Kirsh SR, Alexander JA, Lowery JC. Fostering implementation of health services research findings into practice: a consolidated framework for advancing implementation science. Implement Sci. 2009;4(1):50. 19664226 10.1186/1748-5908-4-50 PMC2736161

[22] Hübsch C, Clarenbach C, Chadwick P, Peterer M, Beckmann S, Naef R, et al. Acceptability, appropriateness and feasibility of a nurse-led integrated care intervention for patients with severe exacerbation of COPD from the healthcare professional’s perspective - a mixed method study. Int J Chron Obstruct Pulmon Dis. 2023;18:1487–97. 37489242 10.2147/COPD.S404712 PMC10363352

[23] Dresing T, Pehl T. Praxisbuch Interview, Transkription & Analyse: Anleitungen und Regelsysteme für qualitativ Forschende. 8. Auflage. Marburg: Eigenverlag; 2018.

[24] Schreier M. Qualitative Content Analysis in Practice. London. California. New Delhi. Singapore: Sage; 2012.

[25] MAXQDA | Die #1 Software für Qualitative & Mixed-Methods-Forschung. MAXQDA. Verfügbar unter: https://www.maxqda.com/de/. [Zitiert 20. Januar 2025]

[26] Leyenaar JK, Arakelyan M, Acquilano SC, Gilbert TL, Craig JT, Lee CN, et al. I-CARE: feasibility, acceptability, and appropriateness of a digital health intervention for youth experiencing mental health boarding. J Adolesc Health. 2023;72(6):923–32. 36870901 10.1016/j.jadohealth.2023.01.015

[27] Ellis S, Geana M, Griebling T, McWilliams C, Gills J, Stratton K, et al. Development, acceptability, appropriateness and appeal of a cancer clinical trials implementation intervention for rural- and minority-serving urology practices. Trials. 2019;20(1):578. 31590694 10.1186/s13063-019-3658-z PMC6781342

[28] Nilsen P, Seing I, Ericsson C, Birken SA, Schildmeijer K. Characteristics of successful changes in health care organizations: an interview study with physicians, registered nurses and assistant nurses. BMC Health Serv Res. 2020;20(1):147. 32106847 10.1186/s12913-020-4999-8 PMC7045403

[29] Cain CL, Surbone A, Elk R, Kagawa-Singer M. Culture and Palliative Care: Preferences, Communication, Meaning, and Mutual Decision Making. J Pain Symptom Manage Mai. 2018;55(5):1408–19. 10.1016/j.jpainsymman.2018.01.007 29366913

[30] Craig P, Dieppe P, Macintyre S, Michie S, Nazareth I, Petticrew M, Medical Research Council Guidance. Developing and evaluating complex interventions: the new Medical Research Council guidance. BMJ. 2008;337:a1655. doi: 10.1136/bmj.a1655. 10.1136/bmj.a1655 PMC2769032 18824488

[31] Arain M, Campbell MJ, Cooper CL, Lancaster GA. What is a pilot or feasibility study? A review of current practice and editorial policy. BMC Med Res Methodol. 2010;10(1):67. 20637084 10.1186/1471-2288-10-67 PMC2912920

[32] Teresi JA, Yu X, Stewart AL, Hays RD. Guidelines for designing and evaluating feasibility pilot studies. Med Care. 2022;60(1):95–103. 34812790 10.1097/MLR.0000000000001664 PMC8849521

