# Supplementary material for: A complex intervention to support the use of sedative drugs in specialist palliative care: results from the iSedPall pilot study
Source: BMC Palliat Care. 2026 May 5;25:128. doi: 10.1186/s12904-026-02112-1 (PMC13147889; doi:10.1186/s12904-026-02112-1)
Supplement: Supplementary file 1 — Supplementary Material 1. [file 12904_2026_2112_MOESM1_ESM.pdf]

Supplementary 1. Questionnaire for physicians.

Note: This is for illustrative purposes only. The original items have been translated to English but have not been validated in English yet.

**1. Participants` characteristics**	
To which occupational group do you currently belong?	○ physician<br>○ nurse<br>○ other profession
In which specialized palliative care setting do you work?	○ inpatient<br>○ homecare
How long have you been working in palliative care overall?	○ > 12 months<br>○ 1-4 years<br>○ at least 5 years
To which age category do you belong?	○ 16-25 years<br>○ 26-35 years<br>○ 36-45 years<br>○ 46-55 years<br>○ > 55 years
**2. Outcome indicator** (based on HePCos [17])	
I prescribe potentially sedative drugs.	○ not part of my current job<br>○ less than once a month<br>○ less than once a week<br>○ at least once a week
When prescribing potentially sedative medications, I feel…	○ unsure<br>○ somewhat unsure<br>○ somewhat sure<br>○ very sure
I evaluate the effect of potentially sedative drugs on the consciousness of patients.	○ not part of my current job<br>○ less than once a month<br>○ less than once a week<br>○ at least once a week
When assessing the effect of potentially sedative medications on patients' consciousness, I feel…	○ unsure<br>○ somewhat unsure<br>○ somewhat sure<br>○ very sure
When patients have reduced consciousness, I assess whether this is likely to be a result of medication.	○ not part of my current job<br>○ less than once a month<br>○ less than once a week<br>○ at least once a week
When assessing whether patients' reduced consciousness is a result of medication, I feel…	○ unsure<br>○ somewhat unsure<br>○ somewhat sure<br>○ very sure
I determine the medication for intentional sedation.	○ not part of my current job<br>○ less than once a month<br>○ less than once a week<br>○ at least once a week
When determining the medication for intentional sedation, I feel…	○ unsure<br>○ somewhat unsure<br>○ somewhat sure<br>○ very sure
Before performing intentional sedation, I explain this procedure to the patient or, if the patient is unable to give consent, to their legal representative.	○ not part of my current job<br>○ less than once a month<br>○ less than once a week<br>○ at least once a week
When informing the patient or (if the patient is unable to give consent) their legal representative about intentional sedation, I feel…	○ unsure<br>○ somewhat unsure<br>○ somewhat sure<br>○ very sure
I document my medical decisions and measures in connection with intentional sedation.	○ not part of my current job<br>○ less than once a month<br>○ less than once a week<br>○ at least once a week
When documenting my medical decisions and measures in connection with intentional sedation, I feel…	○ unsure<br>○ somewhat unsure

	○ somewhat sure<br>○ very sure
I am confronted with ethical challenges in the context of intentional sedation.	○ not part of my current job<br>○ less than once a month<br>○ less than once a week<br>○ at least once a week
When confronted with ethical challenges in the context of intentional sedation, I feel…	○ unsure<br>○ somewhat unsure<br>○ somewhat sure<br>○ very sure
**Additional questions on sedation practice**	
Have you read the SedPall study group's recommendations on the use of sedative drugs?	○ yes<br>○ no
I am aware of when intentional sedation is indicated.	○ does not apply<br>○ does not really apply<br>○ partly/partly<br>○ applies somewhat<br>○ applies completely
I have sufficient experience to perform intentional sedation.	○ does not apply<br>○ does not really apply<br>○ partly/partly<br>○ applies somewhat<br>○ applies completely
**3. Process evaluation** (based on the CFIR [21])	
I am familiar with the iSedPall materials.	○ strongly disagree<br>○ somewhat disagree<br>○ neither agree nor disagree<br>○ somewhat agree<br>○ strongly agree<br>○ i cannot say
I like the design of the iSedPall materials provided.	see above
The iSedPall materials are used as standard for the (planned) use of potentially sedative drugs.	see above
I feel confident when using iSedPall materials.	see above
The iSedPall materials help me to meet the needs of patients and their families.	see above
The iSedPall materials developed are consistent with my personal values and standards.	see above
In my opinion, there is a great need for iSedPall materials.	see above
The use of iSedPall materials has changed the way we handle potentially sedative drugs and intentional sedation in our organization.	see above
In my organization, new ideas are generally welcomed and used to make improvements.	see above
My supervisor supported me in using the iSedPall materials.	see above
The iSedPall materials can be integrated into the existing workflows and practices in my work environment.	see above
The iSedPall materials developed are consistent with the values and standards within my organization.	see above
I had sufficient time to familiarize myself with the iSedPall materials.	see above
The training videos at the start of the pilot phase helped me understand the objectives and content of the iSedPall materials.	see above
What could have been done better in the training videos?	open question
If I had any questions about the iSedPall materials, the people in charge on site were always available to help me.	see above
The iSedPall materials should remain available and continue to be used in my organization after the project has ended.	see above
What would have been helpful in order to make better use of the iSedPall materials in everyday clinical practice?	open question
**4. Scope of the tools` usage**	
I have already used the following iSedPall materials (multiple answers possible):	○ screening tool<br>○ warning list<br>○ recommendations regarding drug doses<br>○ information sheets for patients and legal representatives

	○ checklist for physicians on information provision<br>○ handout for informal caregivers of sedated patients<br>○ documentation templates<br>○ ethical screening tool<br>○ analyses of ethically challenging situations<br>○ checklists for deliberation<br>○ informational brochure<br>○ no material used<br>○ comments
I would like to continue using the following iSedPall materials after the project has ended (multiple answers possible):	see above
**5. Primary feasibility outcomes** (see Weiner et al. [20])	
**6. Final question**	
Is there anything else you would like to tell us?	open question
