# Supplementary material for: A complex intervention to support the use of sedative drugs in specialist palliative care: results from the iSedPall pilot study
Source: BMC Palliat Care. 2026 May 5;25:128. doi: 10.1186/s12904-026-02112-1 (PMC13147889; doi:10.1186/s12904-026-02112-1)
Supplement: Supplementary file 2 — Supplementary Material 2. [file 12904_2026_2112_MOESM2_ESM.pdf]

Supplementary 2. Interview guide for focus group discussions.

Note: This is for illustrative purposes only. The original focus group discussion took place in German.

<u>Notes</u>

- Participants of pilot centres:
- Participants of research team:
- Moderator:
- Observer:

**Introduction**	
1. Welcome and introduction of the scientific staff members of the iSedPall project who are present<br>2. Informed consent<br>3. Questionnaire regarding socio-demographic data and level of professional experience<br>4. Purpose and aims of this focus group discussion<br>5. Explanation of conducting focus groups and conversation rules<br>6. Explanation of procedure	
Start of audio recording	
**Focus group discussion**	
7. Introduction round of all participants<br>8. Input (visual presentation of intervention elements)	
Main question	*Potential follow-up*
**9. If you imagine that another palliative care facility, inpatient or homecare, would like to use the material: What would you tell them about your experience with the iSedPall material?**	• Would you recommend introducing the material? If so, why? If not, why not?<br>• How do you feel about the material provided? What is going well? What is difficult?<br>• What different perspectives are there in your team (in terms of the material)?<br>• What differences are there between the various professions?<br>• What should be considered when introducing the material? What are the factors that promote the introduction of the material? What are the factors that may hinder it?<br>• Can the iSedPall materials be used without explanation?<br>• Should an introduction be provided, or is the material self-explanatory?<br>• What role did the training videos play for you?

<table>
<tr><td></td><td>Can you describe how the material was integrated into existing processes? What was needed to achieve this?
What was easy to integrate into the work processes, and what was difficult?
Does everyone see this in the same way?
What else is important?</td></tr>
<tr><td>10. With regard to the iSedPall material, many participants in the online survey responded that their use of sedative drugs and sedation has changed. How has this change manifested itself in your work context?</td><td>Which changes do you perceive as positive and which as negative?
How have meetings with each other changed?
How has the way you interact with patients changed?
How has documentation practice changed?
Has this resulted in time savings or additional work?
How has the use of sedative drugs changed?</td></tr>
<tr><td>11. When you think about the individual materials, which ones do you find particularly relevant to your own work, and which ones less so?</td><td>Which tools do you find helpful?
Which ones were not helpful?
What about the different professions?
Which materials are always important for you to work with (depending on the case), and which ones do you consider sufficient to simply be familiar with?
Do you recognize these tools from your everyday work? (Show initial input)
Why did you use certain materials and not others?
Can you explain why, to our knowledge, based on the questionnaire survey and previous discussions, the ethics material was hardly used or not used at all?</td></tr>
<tr><td>12. In your opinion, what adjustments or changes are necessary to ensure that the materials can be used effectively?</td><td>What could be improved?
Questions about individual tools
Are there any aspects that we have not considered and that you feel are missing? Which ones? E.g.
- Emergency situations;
- Digital use (or is it better to remain analog?);
- Profession-specific adjustments and tools (to what extent? What might these look like? E.g. screening tool for nursing care?)</td></tr>
<tr><td colspan="2">Conclusion</td></tr>
<tr><td colspan="2">13. Brief summary of the topics discussed by the moderator
14. Is there anything else you would like to say on this topic that we have not addressed so far?
15. Thanks and farewell</td></tr>
</table>
