# Supplementary material for: A complex intervention to support the use of sedative drugs in specialist palliative care: results from the iSedPall pilot study
Source: BMC Palliat Care. 2026 May 5;25:128. doi: 10.1186/s12904-026-02112-1 (PMC13147889; doi:10.1186/s12904-026-02112-1)
Supplement: Supplementary file 3 — Supplementary Material 3. [file 12904_2026_2112_MOESM3_ESM.docx]

**Supplementary file 3. Process evaluation – Frequency distribution**

| Item | **`The iSedPall materials can be integrated into the existing workflows and practices in my work environment.`** | | |
| --- | --- | --- | --- |
|  |  | Frequency | Percentage |
|  | Completely disagree | 1 | 4.2%* |
|  | Rather Disagree | 6 | 25.0%* |
|  | Indifferent | 7 | 29.2%* |
|  | Rather agree | 7 | 29.2%* |
|  | Completely agree | 3 | 12.5%* |
|  | No opinion | 6 | 20.0% |
|  | Total | 30 |  |
|  | Missing | 3 |  |
|  | **`The iSedPall materials are used as standard for the (planned) use of potentially sedative drugs.`** | | |
|  | Completely disagree | 0 | 0.0%* |
|  | Rather Disagree | 5 | 21.7%* |
|  | Indifferent | 2 | 8.7%* |
|  | Rather agree | 15 | 65.2%* |
|  | Completely agree | 1 | 4.3%* |
|  | No opinion | 7 | 23.3% |
|  | Total | 30 |  |
|  | Missing | 3 |  |
|  | **` The training videos at the start of the pilot phase helped me understand the objectives and content of the iSedPall materials.`** | | |
|  | Completely disagree | 4 | 14.8%* |
|  | Rather Disagree | 3 | 11.1%* |
|  | Indifferent | 1 | 3.7%* |
|  | Rather agree | 10 | 37.0%* |
|  | Completely agree | 9 | 33.3%* |
|  | No opinion | 3 | 10.0% |
|  | Total | 30 |  |
|  | Missing | 3 |  |

***Note***. * = `no opinion` option excluded.
